# Relationship between *cyclooxygenase 8473T>C *polymorphism and the risk of lung cancer: a case-control study

**DOI:** 10.1186/1471-2407-6-70

**Published:** 2006-03-17

**Authors:** Jung Min Park, Jin Eun Choi, Myung Hwa Chae, Won Kee Lee, Sung Ick Cha, Ji-Woong Son, Chang Ho Kim, Sin Kam, Young Mo Kang, Tae Hoon Jung, Jae Yong Park

**Affiliations:** 1 Cancer Research Institute, School of Medicine, Kyungpook National University, Dong In 2Ga 101, Daegu, 700-422, South Korea; 2 Department of Preventive Medicine, School of Medicine, Kyungpook National University, Dong In 2Ga 101, Daegu, 700-422, South Korea; 3 Department of Internal Medicine, School of Medicine, Kyungpook National University, Dong In 2Ga 101, Daegu, 700-422, South Korea; 4 Department of Internal Medicine, Konyang University College of Medicine, Naedong 26, Nonsan, 320-711, Korea

## Abstract

**Background:**

Cyclooxygenase-2 (COX-2) plays an important role in the development of lung cancer. DNA sequence variations in the *COX-2 *gene may lead to altered COX-2 production and/or activity, and so they cause inter-individual differences in the susceptibility to lung cancer. To test this hypothesis, we investigated the association between the *8473T>C *polymorphism in the 3'-untranslated region of the *COX-2 *gene and the risk of lung cancer in a Korean population.

**Methods:**

The *COX-2 *genotypes were determined using PCR-based primer-introduced restriction analysis in 582 lung cancer patients and in 582 healthy controls that were frequency-matched for age and gender.

**Results:**

The distribution of the *COX-2 8473T>C *genotypes was not significantly different between the overall lung cancer cases and the controls. However, when the cases were categorized by the tumor histology, the combined *8473 TC *+ *CC *genotype was associated with a significantly decreased risk of adenocarcinoma as compared with the *8473 TT *genotype (adjusted OR = 0.64; 95% CI = 0.46–0.90, *P *= 0.01). On the stratification analysis, the protective effect of the combined *8473 TC *+ *CC *genotype against adenocarcinoma was statistically significant in the males, older individuals and ever-smokers (adjusted OR = 0.59; 95% CI = 0.39–0.91, *P *= 0.02; adjusted OR = 0.55; 95% CI = 0.33–0.93, *P *= 0.03; and adjusted OR = 0.57; 95% CI = 0.37–0.87, *P *= 0.01, respectively).

**Conclusion:**

These findings suggest that the *COX-2 8473T>C *polymorphism could be used as a marker for the genetic susceptibility to adenocarcinoma of the lung.

## Background

Although cigarette smoking is the major cause of lung cancer, only a fraction of smokers develop lung cancer during their lifetime, and this suggests that genetic factors are of importance in determining an individual's susceptibility to lung cancer [1].

Cyclooxygenase (COX, also known as prostanglandin endoperoxide synthase) is the rate-limiting enzyme for the production of prostaglandins (PGs) from free arachidonic acid. Two isoforms of COX have been identified. COX-1 is constitutively expressed in most normal tissues and it is considered to be a housekeeping enzyme that is responsible for various physiologic functions. In contrast, COX-2 is rarely expressed in normal tissues, but it is rapidly induced in response to cytokines, growth factors and tumor promoters [2. 3]. Overexpression of COX-2 has been reported in a variety of human malignancies, including lung cancer [4-6]. Up-regulation of COX-2 can promote tumor growth and metastasis by stimulating cell proliferation, angiogenesis, and invasiveness in addition to inhibiting apoptosis and immune surveillance [7-10]. Moreover, a number of epidemiologic studies have recently suggested that COX-2 inhibitor may have a protective effect on the development of lung cancer [11,12]. Taken together, these observations indicate that COX-2 plays an important role in the development of lung cancer. In addition, several studies have shown that the frequency and level of COX-2 up-regulation was higher in adenocarcinoma (AC) than in squamous cell carcinoma (SCC), and small cell carcinoma (SM) showed virtually negligible expression [6,13,14]. This suggests that the *COX-2 *gene may have a pronounced association with the development of AC.

Single nucleotide polymorphisms are the most common form of human genetic variation, and they may contribute to the individual susceptibility to cancer. Many studies have demonstrated that some of these variants affect either the expression or activities of enzymes; therefore, they are associated with the risk of cancer [15,16]. Thus, we hypothesized that functional SNPs in the *COX-2 *gene may have an impact on COX-2 expression or activity, and so they modulate the susceptibility to lung cancer. To test this hypothesis, a case-control study was conducted to evaluate the association between *COX-2 *polymorphisms and the risk of lung cancer. Among the identified *COX-2 *polymorphisms [17-19], we have focused on the -*1186T>G *and -*765G>C *in the promoter region and the *8473T>C *in the 3'-untranslated region (3'-UTR) since these polymorphisms in the transcription or posttranscriptional regulatory regions have been reported to be associated with various human diseases [17-21]. In the present study, we evaluated with the association of the *8473T>C *polymorphism with lung cancer because the -*1186T>G *and -*765G>C *polymorphisms were not detected in a preliminary study that consisted of 27 lung cancer cases and 27 healthy controls.

## Methods

### Study population

This case-control study included 582 lung cancer patients and 582 healthy controls. The details of the study population have been described elsewhere [22-24]. In brief, the eligible cases included all the patients who were newly diagnosed with primary lung cancer between January 2001 and June 2002 at Kyungpook National University Hospital, Daegu, Korea. There were no age, gender, histological or stage restrictions, but patients with a prior history of cancers were excluded from this study. The cases included 270 (46.4%) SCCs, 205 (35.2%) ACs, 97 (16.7%) SMs, and 10 (1.7%) large cell carcinomas. The demographics and clinical characteristics of the cases were consistent with those of a nationwide lung cancer survey conducted by the Korean Academy of Tuberculosis and Respiratory Disease in 1998 [25]. The control subjects were randomly selected from a pool of healthy volunteers who visited the general health check-up center at Kyungpook National University Hospital during the same period. The control subjects were frequency matched (1:1) to the cancer cases based on gender and age (± 5 years). All the cases and the controls were ethnic Koreans and they resided in Daegu City or the surrounding regions. A detailed questionnaire was completed for each patient and control by a trained interviewer. The questionnaire included information on the average number of cigarettes smoked daily and the number of years the subjects had been smoking. For smoking status, a person who had smoked at least once a day for > 1 year in his or her lifetime was regarded as a smoker. A former smoker was defined as one who had stopped smoking at least 1 year before diagnosis in the case of patients and 1 year before the date signed on an informed consent for blood sample collection in the case of controls. Cumulative cigarette dose (pack-years) was calculated using the following formula: pack-years = (packs per day) × (years smoked). This study was approved by the institutional review board of the Kyungpook National University Hospital, and written informed consent was obtained from each participant.

### COX-2 genotyping

Genomic DNA was extracted from peripheral blood lymphocytes by proteinase K digestion and phenol/chloroform extraction. The *COX-2 8473T>C *genotype was determined using PCR-based primer-introduced restriction analysis (PIRA) as described by Hu et al. [26]. The PCR products were digested overnight with 10 units of *Bcl*I (New England BioLabs, Inc., Beverly, MA, USA) at 50°C. The digested PCR products were resolved on 6% acrylamide gel and stained with ethidium bromide for visualization under UV light. To ensure quality control, the genotyping analysis was performed "blind" with respect to case/control status. About 10% of the samples were randomly selected to be genotyped again by a different author, and the results were 100% concordant. To confirm the genotyping results, selected PCR-amplified DNA samples (n = 2, respectively, for each genotype) were examined by DNA sequencing, and the results were also 100% concordant.

### Statistical analysis

Cases and controls were compared using the Student's t-test for continuous variables and the χ^2 ^test for categorical variables. Hardy-Weinberg equilibrium was tested with a goodness-of-fit χ^2 ^test with one degree of freedom for the comparing the observed genotype frequencies with the expected genotype frequencies among the subjects. Unconditional logistic regression analysis was used to calculate odds ratios (ORs) and 95% confidence intervals (CIs), with adjustment for the possible confounders (gender as a nominal variable; age and pack-years of smoking, as continuous variables). Multivariate logistic regression analyses were performed to analyze the association between the genotypes and the risk of lung cancer after stratification according to age (median age), gender, smoking status, cigarette consumption (median pack-years), and histological types of lung cancer. In case of subgroup analysis for AC, the smoking status was categorized into two groups: never-smokers and ever-smokers (former- and current-smokers) since the number of former smokers in the AC cases was small (n = 29). All analyses were performed using Statistical Analysis Software for Windows, version 8.12 (SAS institute, Gary, NC, USA).

## Results

The demographics of the cases and controls enrolled in this study are shown in Table 1. There were no significant differences between the cases and controls for the mean age or gender distribution, and this suggested that the matching based on these two variables was adequate. The case group had a higher prevalence of current smokers than did the controls (*P *< 0.001), and the number of pack-years in the smokers was significantly higher in the cases than in the controls (40.0 ± 17.7 versus 34.1 ± 17.8 pack-years; *P *< 0.001). These differences were controlled in the later multivariate analyses.

The genotypes and polymorphic 8473C allele frequencies of the *COX-2 8473T>C *polymorphism among the controls and cases are shown in Table 2. The distribution of the genotypes among the controls was in Hardy-Weinberg equilibrium (χ = 0.35, *P *= 0.55). No significant deviation was observed for the distribution of the genotypes between the overall lung cancer cases and the controls. However, when the cases were categorized by the tumor histology, the distribution of the *8473T>C *genotypes among the AC cases differed significantly from that among the controls (*P *= 0.04). The distributions of the genotypes in the SCC and SM groups were not significantly different from that among the controls.

The lung cancer risk related to the *COX-2 8473T>C *genotypes was also shown in Table 2. When the *8473 TT *genotype was used as the reference group, the *8473 TC *and the *8473 CC *genotypes were associated with a non-significant decreased risk for overall lung cancer, respectively [adjusted odds ratio (OR) = 0.87; 95% confidence interval (CI) = 0.68–1.11; and adjusted OR = 0.72; 95% CI = 0.41–1.25, respectively). When the analyses were stratified by the tumor histology, the *8473 TC *genotype and the combined *8473 TC *+ *CC *genotype were associated with a significantly decreased risk of AC, respectively (adjusted OR = 0.64; 95% CI = 0.45–0.91, *P *= 0.01; and adjusted OR = 0.64; 95% CI = 0.46–0.90, *P *= 0.01, respectively). No significant association was found between the *8473T> C *polymorphism and the risk of SCC or SM.

The association between the *COX-2 8473T>C *genotypes and the risk of AC was further examined by stratifying according to gender, age and the smoking status (Table 3). As compared with the *8473 TT *genotype, the combined *8473 TC *+ *CC *genotype was associated with a significantly decreased risk of AC in the males and older individuals (adjusted OR = 0.59; 95% CI = 0.39–0.91, *P *= 0.02; and adjusted OR = 0.55; 95% CI = 0.33–0.93, *P *= 0.03, respectively), whereas it had no significant effect of the risk of AC in the females and younger individuals. When stratified by the smoking status, a significant protective effect of the combined *8473 TC *+ *CC *genotype against AC was observed in the ever-smokers (adjusted OR = 0.57; 95% CI = 0.37–0.87, *P *= 0.01), but not in never-smokers. When the ever-smokers were dichotomized by the median pack-years of smoking, the protective effect of the combined *8473 TC *+ *CC *genotype was similar in lighter smokers and heavier smokers (data not shown).

## Discussion

We investigated the influence of *COX-2 *polymorphisms on the risk of lung cancer by conducting a hospital-based case-control study. The *8473T>C *polymorphism in the 3'-UTR region of the *COX-2 *gene was associated with a significantly decreased risk of AC of the lung. The protective effects were evident in the males, the older individuals and the smokers. These findings suggest that the *COX-2 8473T>C *polymorphism could be used as a marker for the genetic susceptibility to AC of the lung. Of three major histological types of lung cancer, the proportion of AC is increasing world-wide. Thus, identifying the genetic factors that are responsible for the susceptibility to AC is indispensable for establishing novel and efficient ways to prevent this disease.

Because the *COX-2 -1186T>G *and -*765G>C *polymorphisms were not detected in the preliminary study that included 27 healthy controls and 27 lung cancer cases, we analyzed only the association of the *COX-2 8473T>C *polymorphism with the risk of lung cancer. The frequency of the *COX-2 8473C *allele among the healthy controls in the current study was 0.244, which was significantly lower than that (0.535) of healthy Norwegians [18], but it was significantly higher than that (0.187) of healthy Chinese [26].

In the current study, the *COX-2 8473T>C *polymorphism was significantly associated with the risk of AC, but it was not associated with SCC or SM. Although the reasons for the observed histology-specific difference in the risk conferred by the *COX-2 *polymorphism remain to be elucidated, this difference may be attributable to the differences in the pathways of carcinogenesis among the different histological types of lung cancer. Therefore, certain genotypes could confer a greater susceptibility to a particular histological type of lung cancer [22-24,27,28]. Several studies have shown that the frequency and level of COX-2 up-regulation was higher in the ACs than in the SCCs, and the SMs showed a virtually negligible expression [6,13,14]. These observations suggest that the *COX-2 *gene may have a pronounced association with the development of AC, and they are comparable with our finding that the *COX-2 *polymorphism plays an important role in determining the genetic susceptibility to AC.

A few studies have investigated the association between the *COX-2 8473T>C *polymorphism and the risk of lung cancer [18,26]. Campa et al. [18] have reported that the *8473 TC *and the *8473 CC *genotypes were associated with a significantly increased risk of lung cancer in a Norwegian population. In contrast to the Norwegian study, Hu et al. [26] have reported that the combined *8473 TC *+ *CC *genotype was associated with a significantly decreased risk of lung cancer in a Chinese population. In concordance with the Chinese study, the combined *8473 TC *+ *CC *genotype was associated with a significantly decreased risk of AC in our current study. Although it is hard to decipher the reasons for the oppositely directed association of the *COX-2 8473T>C *polymorphism for lung cancer in the different studies, several genetic and environmental factors that are relevant to this polymorphism might have caused the different results. Moreover, since a protective genotype in one population may increase the cancer risk in another population due to other linked polymorphisms that exhibit stronger effects on the susceptibility to cancer, the different genetic background of the study subjects should be also considered. In addition, inadequate study design such as nonrandom sampling, a limited sample size and the pitfalls arising from unknown confounders should be also considered.

Whether the *COX-2 8473T>C *polymorphism itself alters mRNA stability and/or translation efficiency or if it is in linkage disequilibrium with the other functional polymorphisms remains to be investigated. The 3'-UTR region of *COX-2 *contains highly conserved adenosine- and uridine-rich elements that are composed of the Shaw-Kamens sequence (AUUUA), which is also known as AU rich elements [29]. This motif is present within the 3'-UTRs of many protooncogenes and cytokine genes, and it is involved in the regulation of COX-2 production by acting both as a message instability determinant and a translation inhibitory element [30-32]. Therefore, the *8473T>C *polymorphism located within the functional region of 3'-UTR of the *COX-2 *gene may affect the message stability and/or translation efficiency, and so this results in differential COX-2 expression.

Several polymorphisms have been identified in the promoter region of the *COX-2 *gene [17-19]. The -*765G>C *polymorphism, which is located at just 5' to a STAT1 binding site, has been found to influence COX-2 expression and it contributes to the risk of various human diseases [17,21,33]. Although the frequency of carrying the -*765 GC *or *CC *genotype has been reported to be 25% to 50% in Caucasians [17,21,33], the -*765G>C *polymorphism was not detected in the preliminary study that included 27 healthy controls. These samples included 54 chromosomes, and this provides for at least a 95% confident level to detect alleles with frequencies of > 5%. Thus, it is very likely that if this polymorphism exists, it may not play a major role in the genetic susceptibility to lung cancer in the Korean population [34,35]. Kang et al. [19] have recently reported that the -*1186T>G *polymorphism in the *nuclear factor kappa-B *binding region was associated with the risk of bladder cancer in a Korean population. In this study, however, the -*1186T>G *polymorphism was very rare (the frequency of the -*1186G *allele was 1.0%), and this not associated with the risk of lung cancer. In the current study, the -*1186T>G *polymorphism was not detected in the preliminary study that included 27 healthy controls and 27 lung cancer cases, suggesting that this polymorphism may not play a major role in the genetic susceptibility to lung cancer in the Korean population.

During multistage carcinogenesis, COX-2 can enhance tumor invasion and metastasis [7-10]; therefore, the *COX-2 8473T>C *polymorphism may have an influence on the progression of disease. In the present study, however, no significant difference was observed in the genotype distribution according to the stage of lung cancer (data not shown).

## Conclusion

We found that the *8473T>C *polymorphism in the 3'-UTR region of the *COX-2 *gene was associated with the risk of lung cancer, and particularly for the risk of AC. This result indicates that this polymorphism could be used as one marker for defining the risk of lung cancer. However, it is possible that our findings, particularly those findings from stratified analyses, can be attributed to chance because of multiple comparisons and/or the relatively small numbers of subjects in the subgroups. Therefore, additional studies with larger sample sizes will be required to confirm these findings. Future studies on the other *COX-2 *sequence variants and their biologic function are also needed to understand the role of the *COX-2 *polymorphisms in determining the risk of lung cancer. Moreover, since genetic polymorphisms often vary significantly between the different ethnic groups, further studies are warranted to clarify the association of the *COX-2 *polymorphisms with the risk of lung cancer in diverse ethnic populations.

## Competing interests

The author(s) declare that they have no competing interests.

## Authors' contributions

JMP, JEC and MHC carried out a PCR-RFLP assay and participated in interpretation of data. WKL and SK participated in the design of study, and analysis and interpretation of data. SIC, JWS and CHK collected clinical data and helped to draft the manuscript. YMK and THJ participated in the design of study, acquisition of data and interpretation of data. JYP conceived the study and participated in the design of study, analysis and interpretation of data, drafting the article and final approval of this version. All authors read and approved the final manuscript.

## Pre-publication history

The pre-publication history for this paper can be accessed here:


